# Supplementary material for: A mitochondrial pentatricopeptide repeat protein enhances cold tolerance by modulating mitochondrial superoxide in rice
Source: Nat Commun. 2023 Oct 25;14:6789. doi: 10.1038/s41467-023-42269-4 (PMC10600133; doi:10.1038/s41467-023-42269-4)

**Supplementary Information**

**A mitochondrial pentatricopeptide repeat protein enhances cold tolerance by modulating mitochondrial superoxide in rice**

Xiaofeng Zu^1, #^, Lilan Luo^1, #,^*, Zhen Wang^1, #^, Jie Gong^1, 2^, Chao Yang^1^, Yong Wang^3^, Chunhui Xu^3^, Xinhua Qiao^4^, Xian Deng^1^, Xianwei Song^1^, Chang Chen^4, 5^, Bao-Cai Tan^3^, Xiaofeng Cao^1,5,6^*

^1^State Key Laboratory of Plant Genomics and National Center for Plant Gene Research, Institute of Genetics and Developmental Biology, Chinese Academy of Sciences, Beijing 100101, China

^2^The Municipal Key Laboratory of the Molecular Genetics of Hybrid Wheat, Institute of Hybrid Wheat, Beijing Academy of Agriculture and Forestry Sciences, Beijing 100097, China

^3^Key Laboratory of Plant Development and Environmental Adaptation Biology, Ministry of Education, School of Life Sciences, Shandong University, Qingdao, 266237, China

^4^National Laboratory of Biomacromolecules, CAS Center for Excellence in Biomacromolecules, Institute of Biophysics, Chinese Academy of Sciences, Beijing 100101, China

^5^University of Chinese Academy of Sciences, Beijing 100049, China

^6^CAS Center for Excellence in Molecular Plant Sciences, Chinese Academy of Sciences, Beijing 100101, China

^#^These authors contributed equally: Xiaofeng Zu, Lilan Luo, and Zhen Wang.

*These authors jointly supervised this work: Lilan Luo (luolilan@genetics.ac.cn) and Xiaofeng Cao (xfcao@genetics.ac.cn).


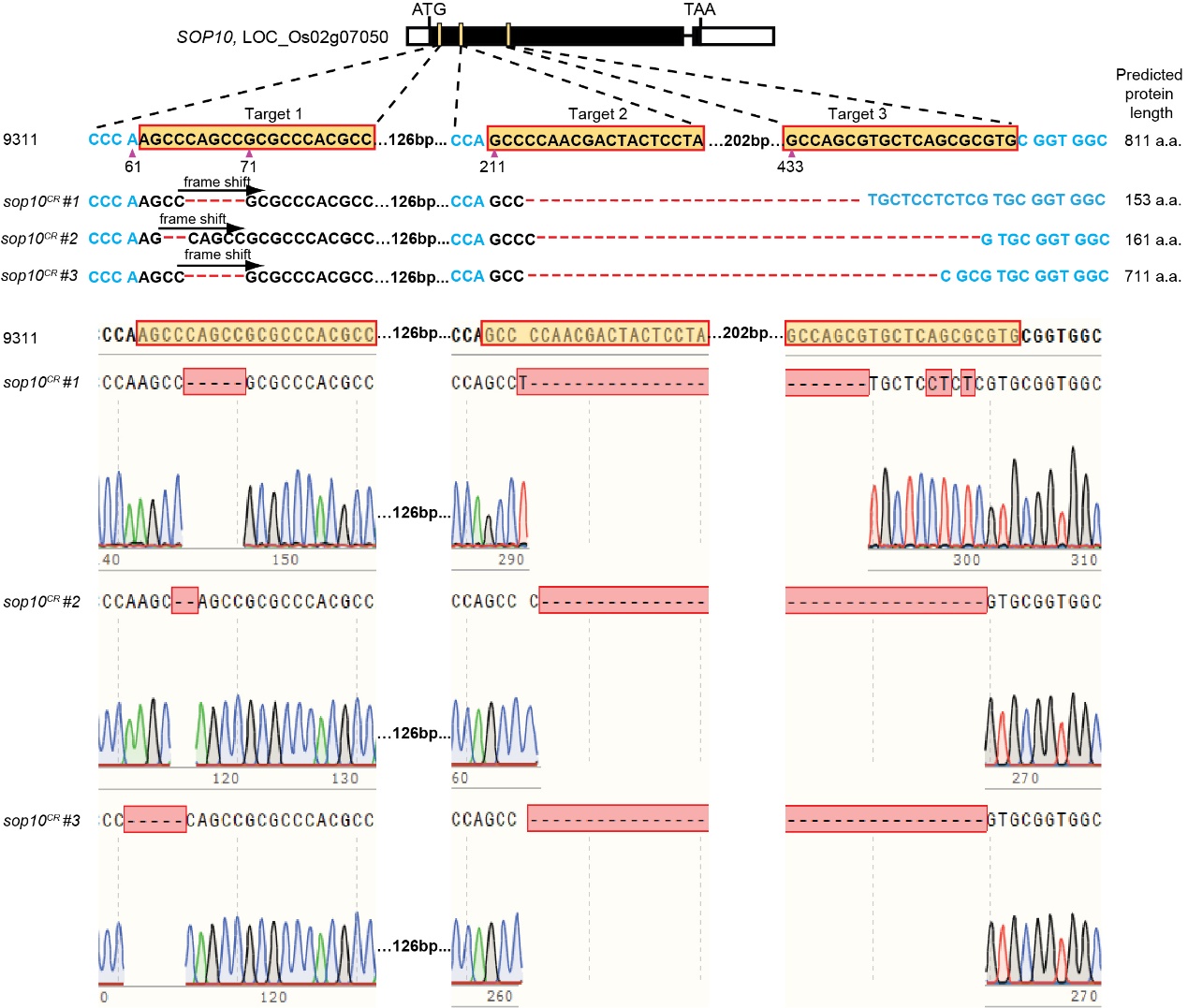


**Supplementary Fig. 1 Genotypes of the *sop10* mutants generated by CRISPR-Cas9.** The sgRNA sequences of CRISPR targeting sites in *SOP10* genomic DNA are highlighted in yellow. The predicted protein lengths of the three independent lines of *sop10* mutants are shown on the right. Genotypes and mutant patterns were confirmed by Sanger sequencing.


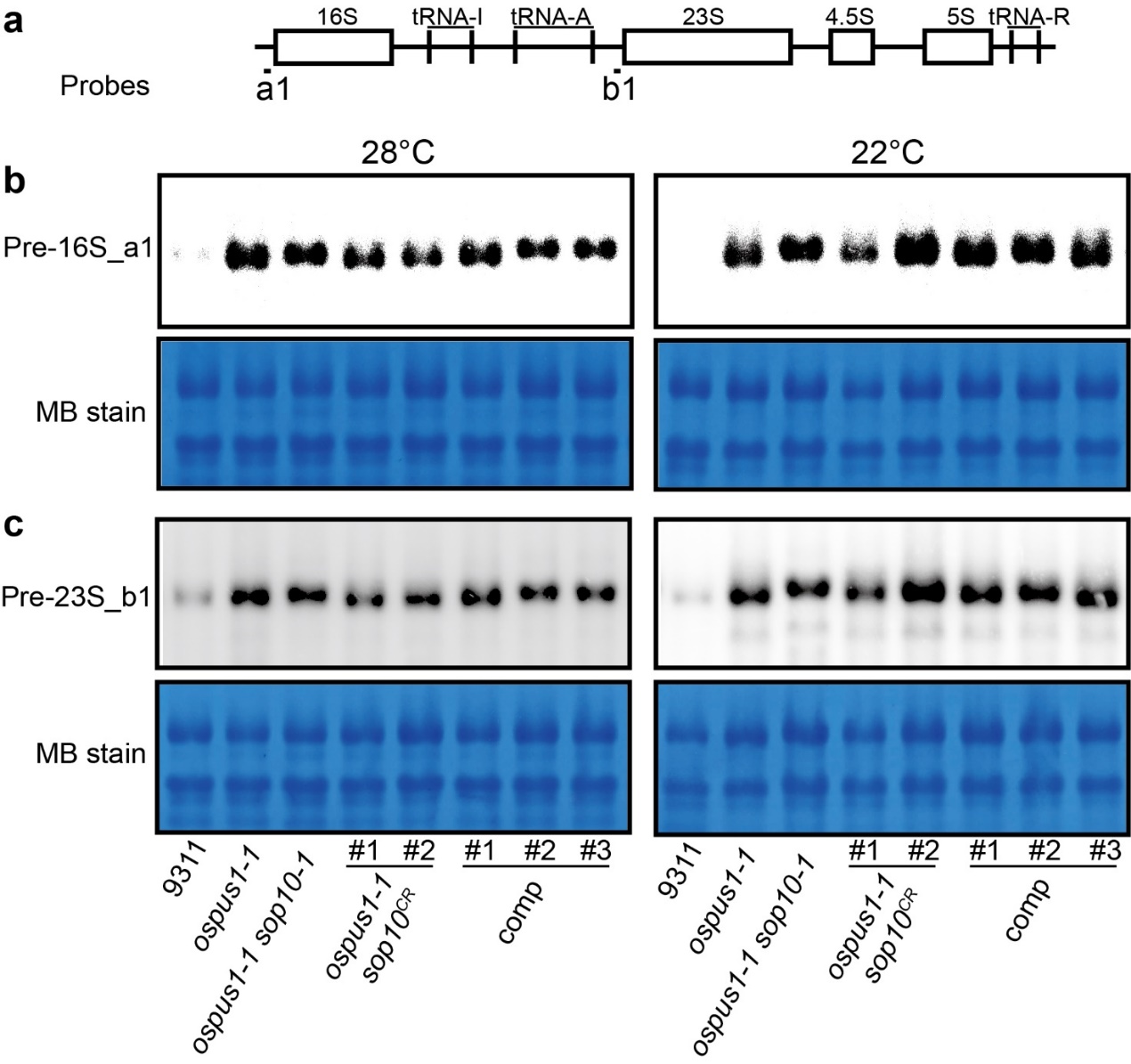


**Supplementary Fig. 2 rRNA processing was not restored** **in *ospus1-1 sop10-1* chloroplasts**. **a**, The structure of the chloroplast rRNA operon, a1 and b1 probes were used for Northern blots. **b** and **c**, The processing patterns of chloroplast 16S and 23S pre-rRNAs. Total RNA was extracted from seedlings of 9311, *ospus1-1*, three suppressors of *ospus1-1* (*ospus1-1 sop10-1* and two *ospus1-1 sop10^CR^* lines), and *ospus1-1 sop10-1* complementation (*comp*) plants grown at 28℃ or 22℃. Probes a1 and b1 were used to detect the pre-16S rRNA and pre-23S rRNA, respectively. Methylene blue (MB) stained blots are shown as a loading control.

**
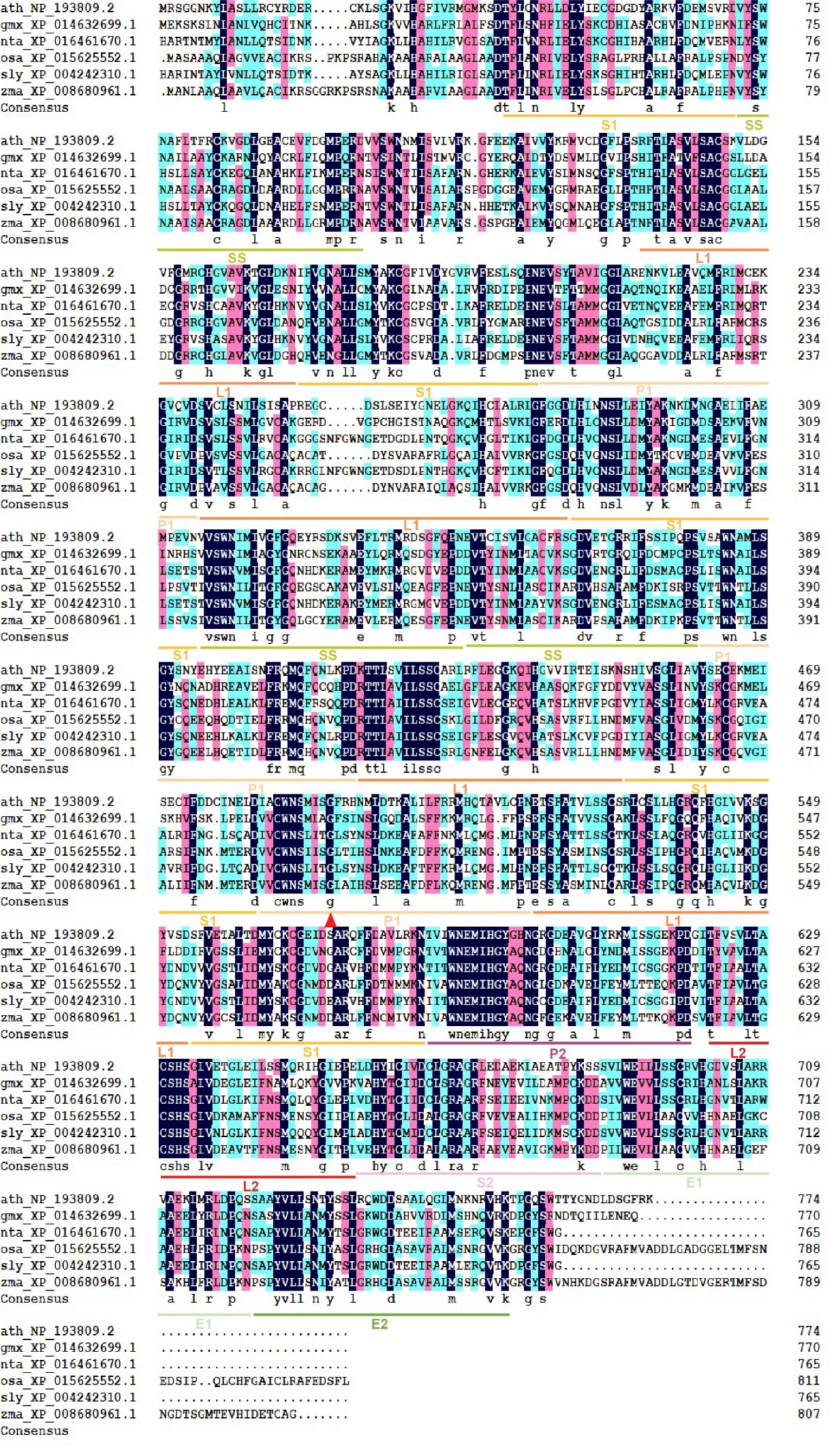
**

**Supplementary Fig. 3 Amino acid sequence alignments of SOP10.** Amino acid sequence alignments of SOP10 from *Arabidopsis thaliana* (*ath*), *Glycine max* (*gmx*), *Nicotiana tabacum* (*nta*), *Oryza sativa* (*osa*), *Solanum lycopersicum* (*sly*), and *Zea mays* (*zma*). Pentatricopeptide repeats (PPRs) in SOP10 are underlined with red lines. Dark, pink, and cyan shading indicate 100%, >75%, and >50% sequence similarity, respectively. The red triangle indicates the mutation site in sop10-1.


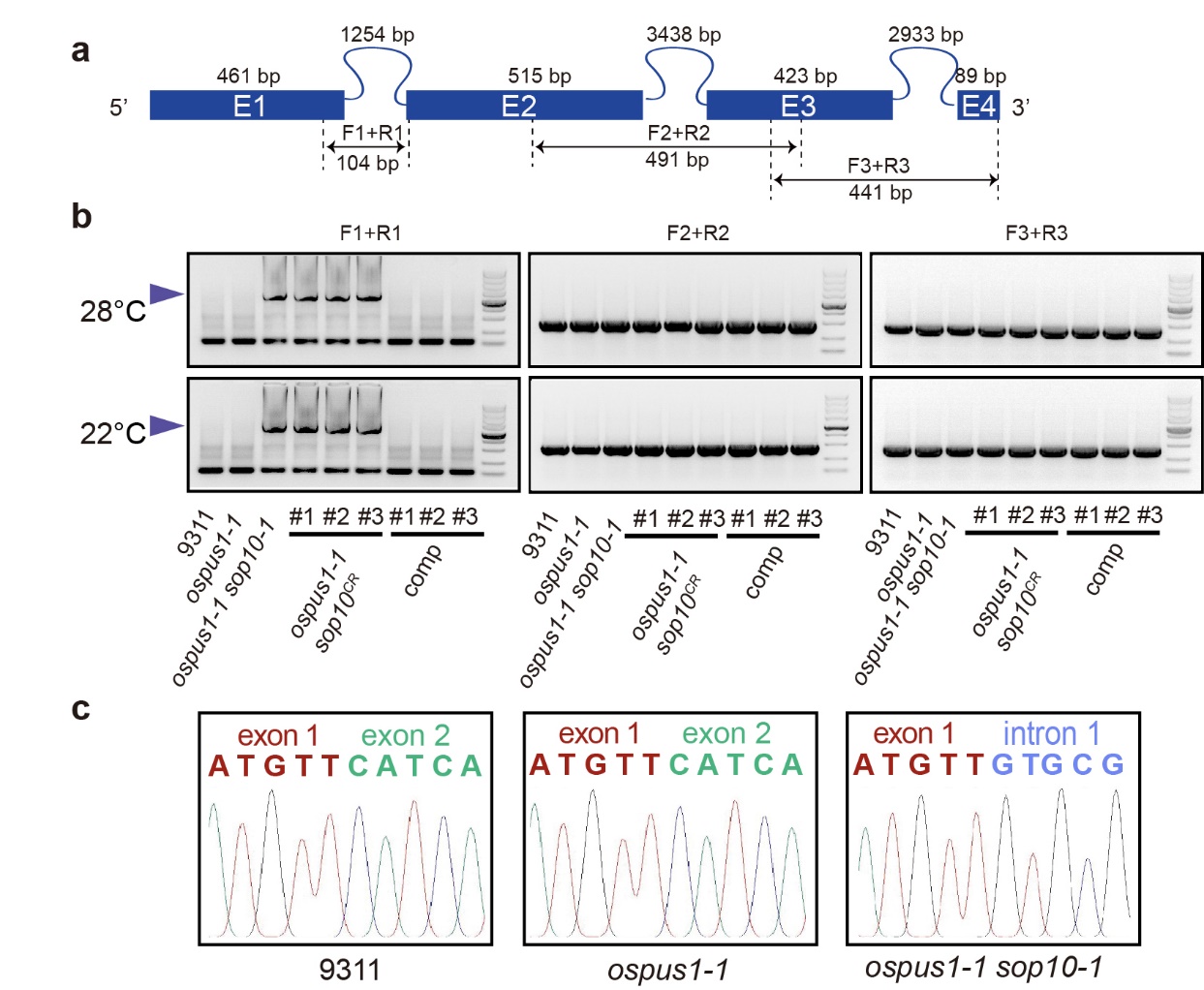


**Supplementary Fig. 4 The first intron of *nad4* is retained in *ospus1-1* *sop10-1* plants. a**, Diagram of *nad4* gene structure. Positions of the primers used for PCR are indicated by black arrows. **b**, Intron retention occurred in *nad4* transcripts in suppressors of *ospus1-1* (*ospus1-1 sop10-1* and three *ospus1-1 sop10^CR^* lines) at 28℃ and 22℃ by RT-PCR. The purple triangle represents the intron-retained *nad4* transcript. **c**, The first intron of *nad4* was retained in *ospus1-1* *sop10-1* plants. Sanger sequencing of RT-PCR products amplified in **b**.


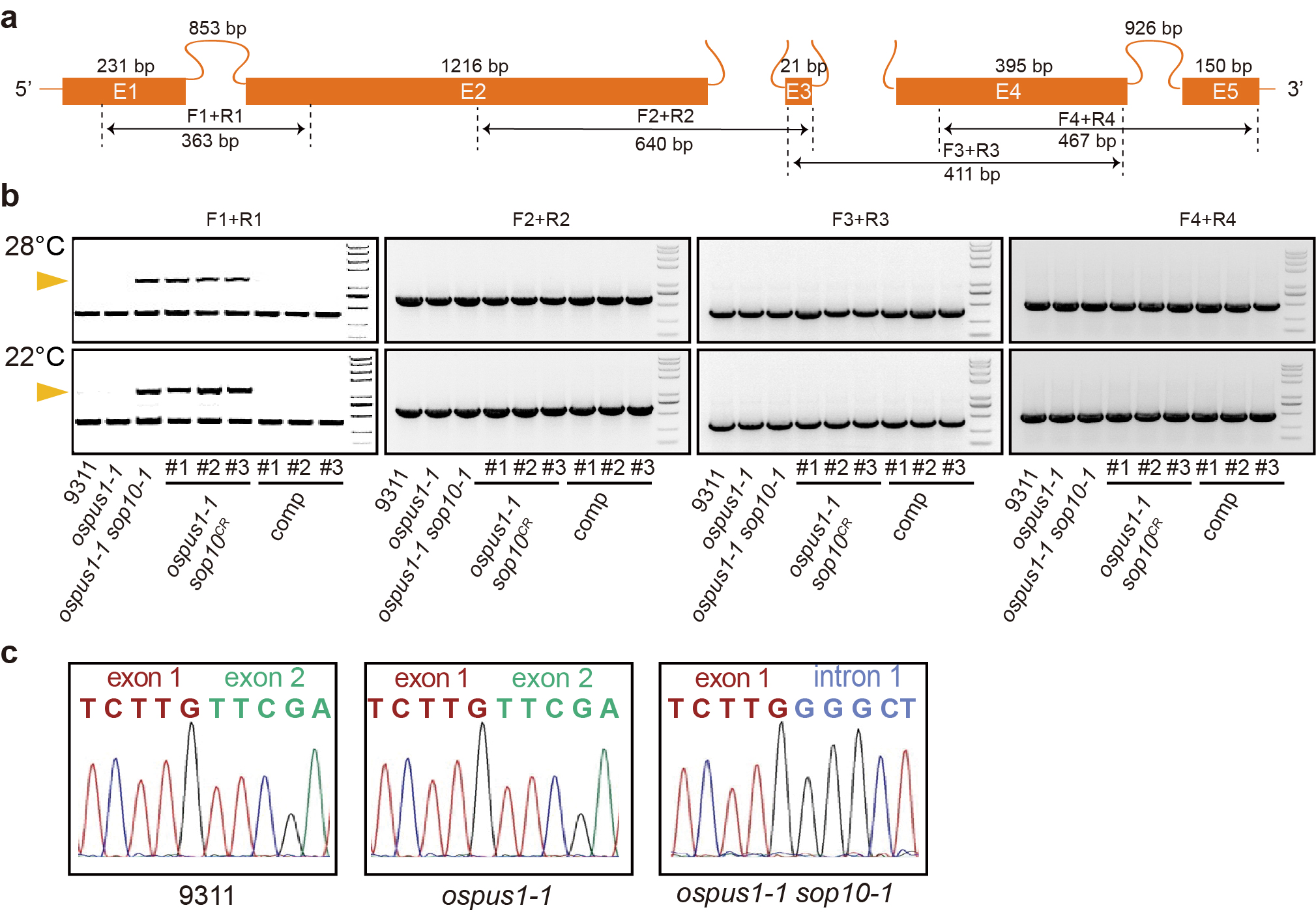


**Supplementary Fig. 5 The first intron of *nad5* is retained in *ospus1-1* *sop10-1* plants. a**, Diagram of *nad5* gene structure. Positions of the primers used for PCR are indicated by black arrows. **b**, Intron retention occurred in *nad5* transcripts in suppressors of *ospus1-1* (*ospus1-1 sop10-1* and three *ospus1-1 sop10^CR^* lines) at 28℃ and 22℃ by RT-PCR. The triangle represents the intron-retained *nad5* transcript. **c**, The first intron of *nad5* was retained in *ospus1-1* *sop10-1* plants. Sanger sequencing of RT-PCR products amplified in **b**.


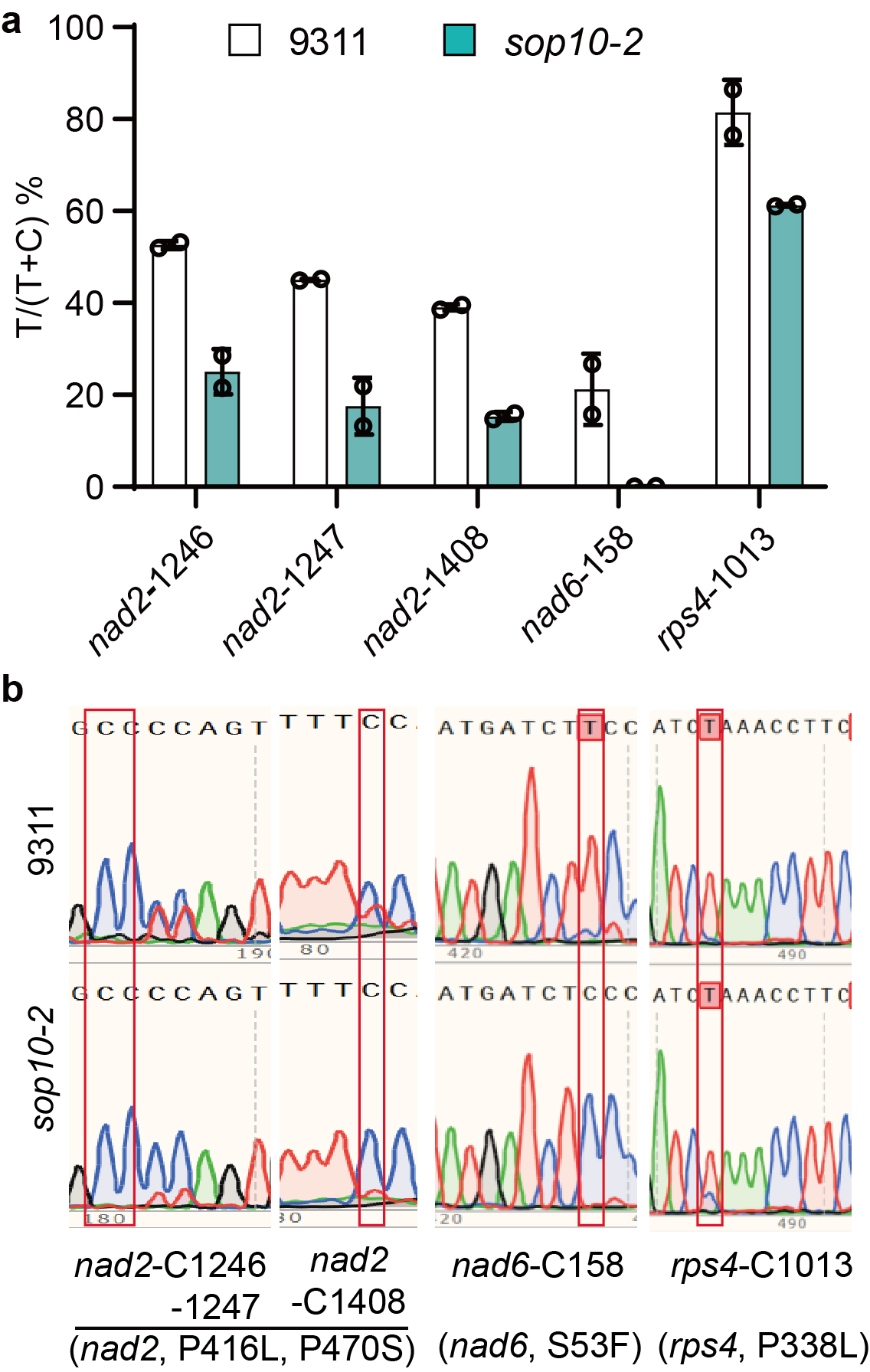


**Supplementary Fig. 6 RNA editing at five mitochondrial sites is defective in the *sop10-2* mutant**. **a**, The editing efficiency is shown by T/(T+C)% in the WT and *sop10-2*. The relevant raw data are reported in Supplementary Data 2. Two independent RNA samples were used to analyze the editing by STS-PCR-seq. The bars show the mean ± S.D. (*n* = 6) of two biological replicates. **b**, The editing sites were confirmed by Sanger sequencing using independent RNA samples from those two used in **a**. The RNA editing sites and the resulting amino acid changes are illustrated below.


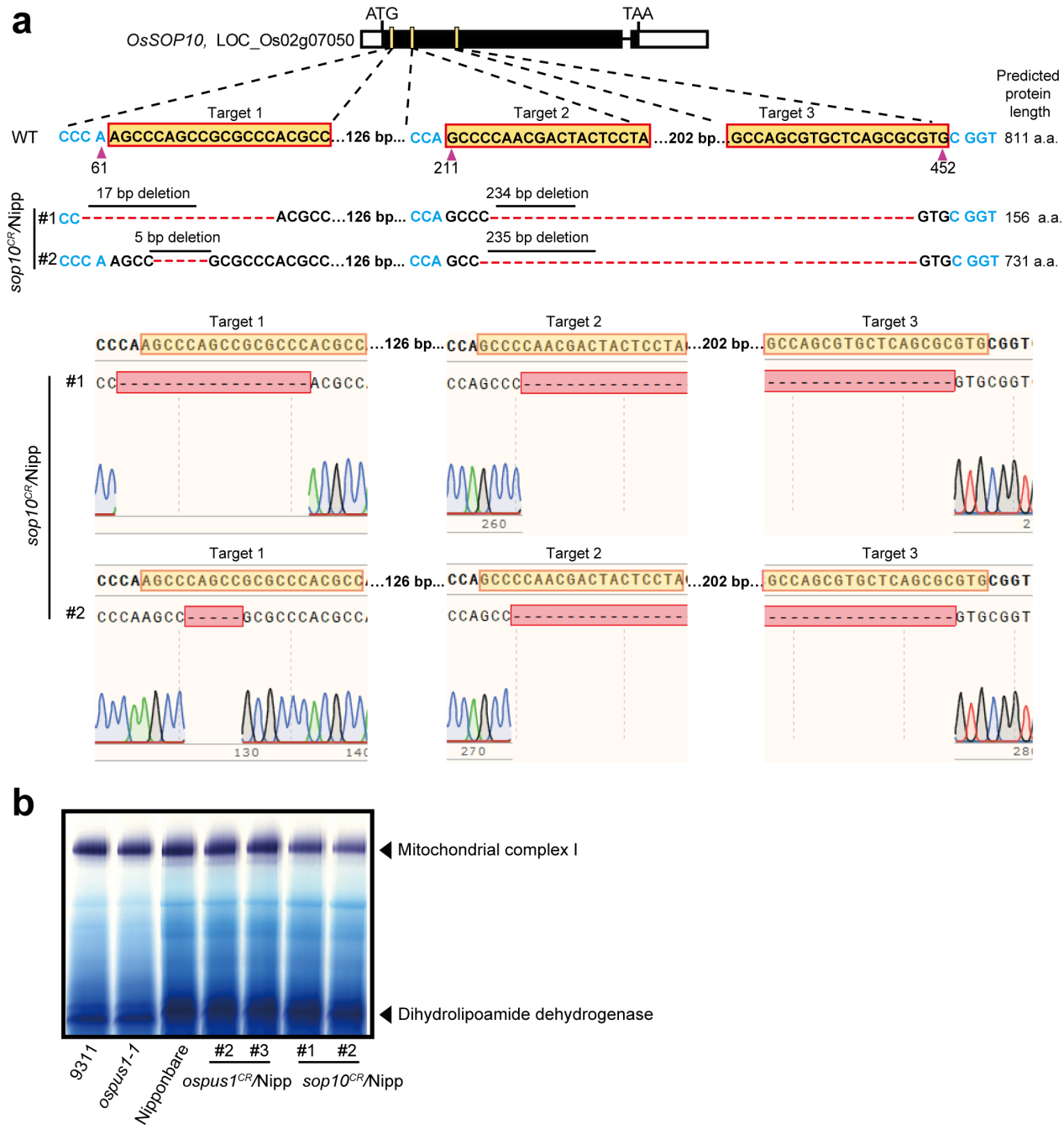


**Supplementary Fig. 7 Genotypes of the *sop10* mutants generated by CRISPR-Cas9. a**, The sgRNA sequences of CRISPR target sites in *SOP10* genomic DNA are highlighted in yellow. **b**, In-gel assay of NADH oxidase capacity. Dihydrolipoamide dehydrogenase activity was used as a loading control.


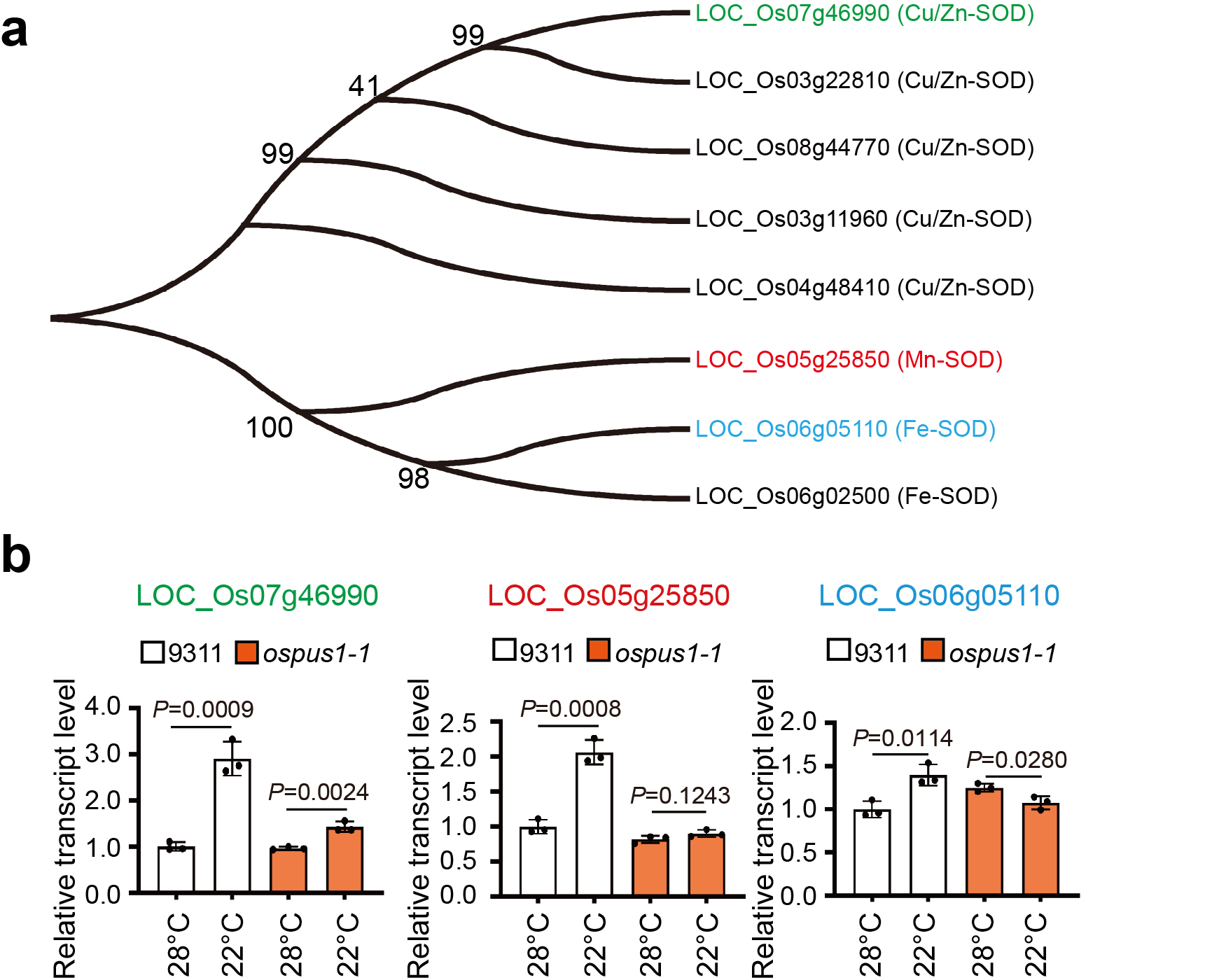


**Supplementary Fig. 8 Expression of SOD genes in leaves is upregulated under cold conditions. a**, Neighbor-joining tree of rice SOD genes was constructed in MEGA7. The numbers represent bootstrap (1000 replicates). The rice SOD gene family contains one Mn-SOD gene, two Fe-SOD genes, and five Cu/Zn-SOD genes. **b**, The relative transcript level of SOD-encoding genes in leaves of 9311 and *ospus1-1* grown at 28℃ or 22℃ quantified by RT-qPCR. Values are means ± S.D. (*n* = 6) of three biological replicates. Differences of SOD gene expression levels in 9311 or *ospus1-1* grown under 28℃ or 22℃ conditions are determined by paired Student’s *t*-test (two-tailed). Exact *P* values are shown.


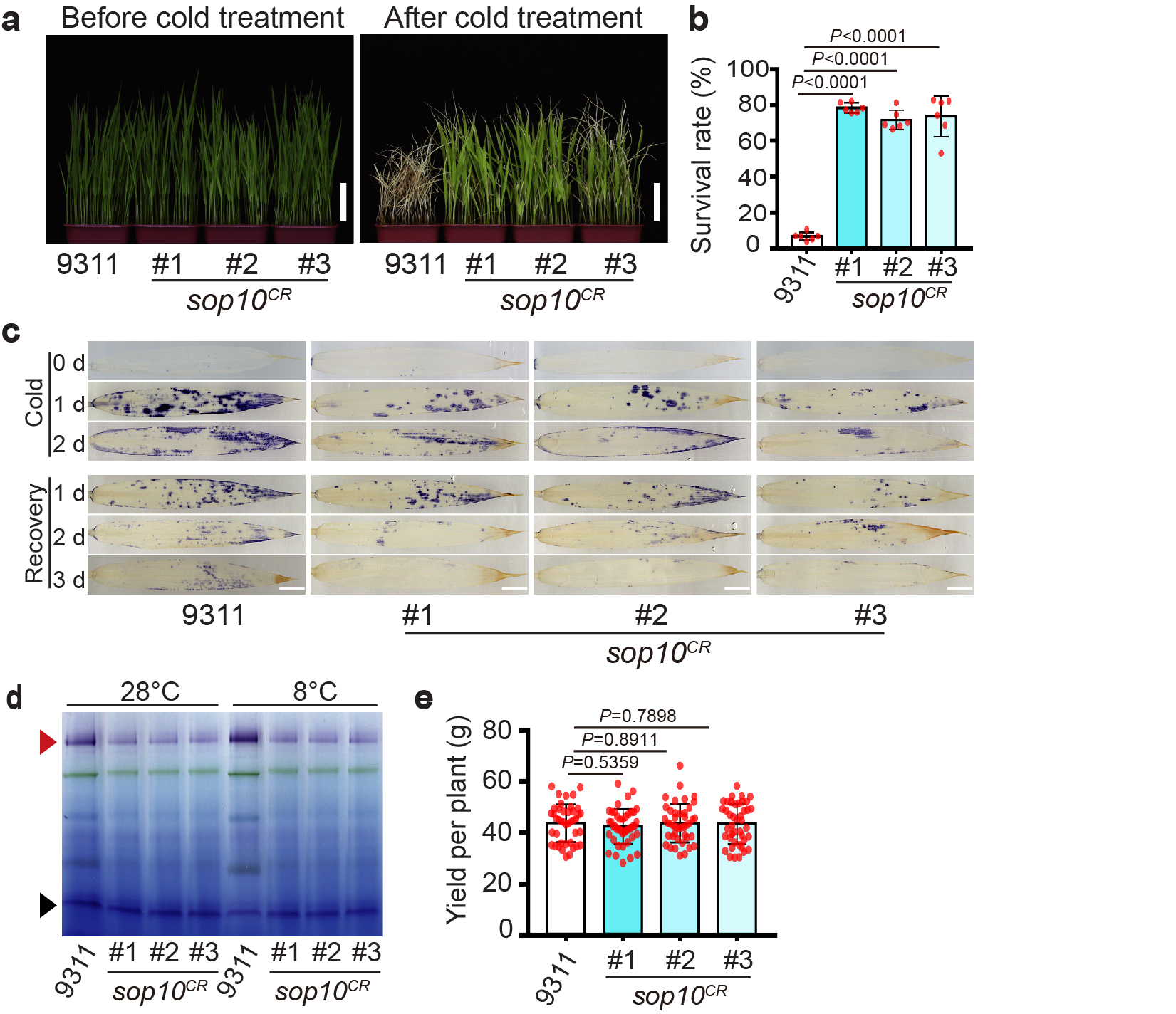


**Supplementary Fig. 9 Cold stress tolerance of *sop10* single mutant plants at the seedling stage.** **a**, Seedlings of 9311 and *sop10^CR^* grown at 28℃for 2 weeks were shifted to 8℃ for 2 days and then allowed to recover at 28℃ for 5 days. Scale bars, 5 cm. **b**, Survival rates of 9311 and *sop10^CR^* seedlings after cold treatment in (**a**). Values are means ± S.D. (*n* = 50 individual plants) of six biological replicates. Differences between mutants and wild type grown under the same conditions are determined by unpaired Student’s *t*-test (two-tailed). Exact *P* values are shown. **c**, NBT staining was used to assess the accumulation of O_2_*^–^* in 9311 and *sop10^CR^* seedlings before and after cold treatment in (**a**), Scale bars, 1 cm. **d**, In-gel assay of NADH oxidase capacity. The dihydrolipoamide dehydrogenase activity was used as a loading control. The red triangle indicates mitochondrial complex I, and the black triangle indicates dihydrolipoamide dehydrogenase. The experiment was repeated two times with the similar results. **e**, Statistical analysis of yield per plant in the field. Values are means ± S.D. (*n* = 42 individual plants). Differences between mutants and wild type grown under the same conditions are determined by unpaired Student’s *t*-test (two-tailed). Exact *P* values are shown.


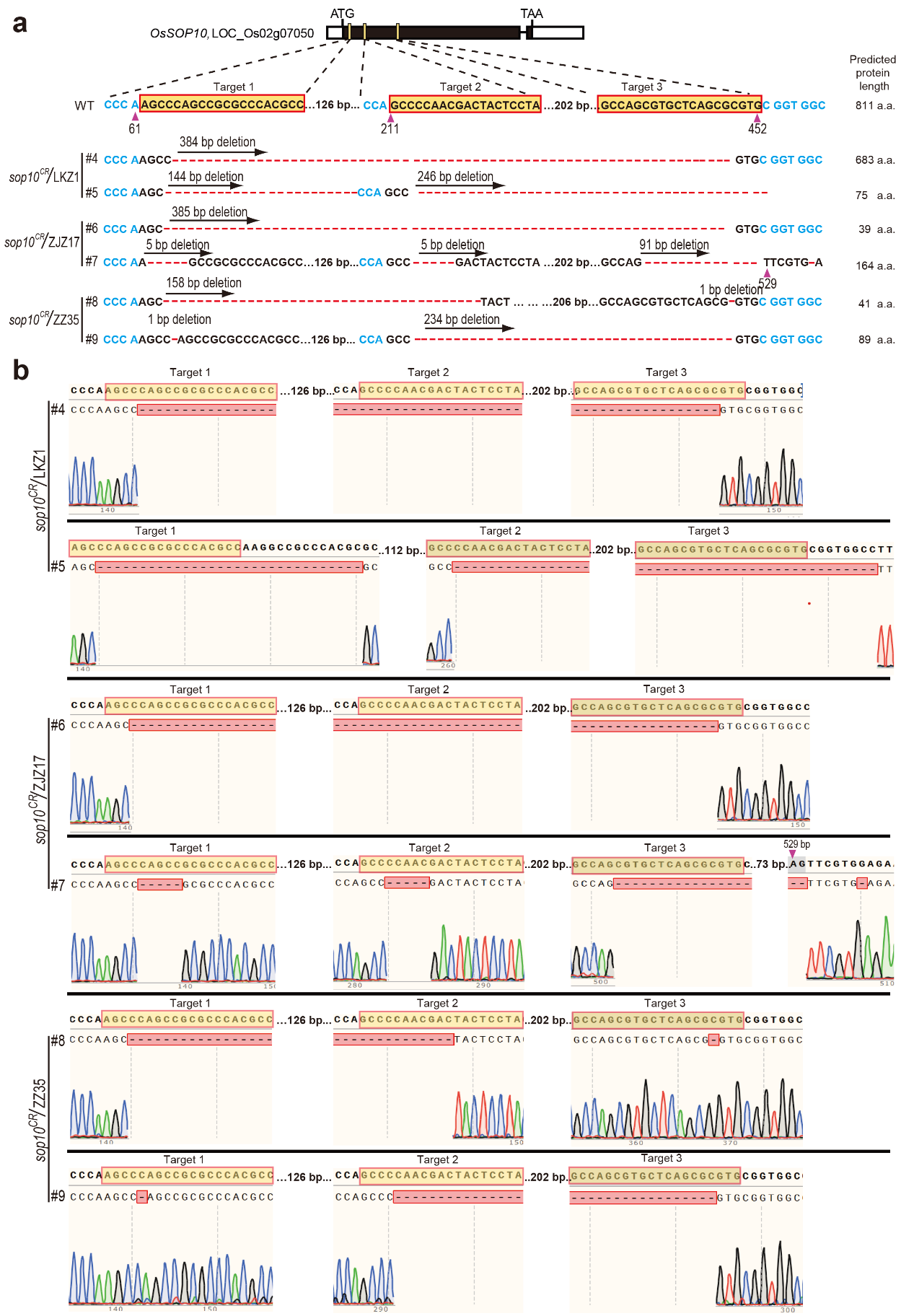


**Supplementary Fig. 10 Genotypes of the *sop10* mutants in various *indica* rice varieties generated by CRISPR-Cas9.** **a**, The sgRNA sequences of CRISPR target sites in the *OsSOP10* genomic DNA are highlighted in yellow. The predicted lengths of the sop10 mutant proteins produced in the LKZ1, ZJZ17, and ZZ35 lines carrying *sop10* mutants are shown on the right. **b**, Sanger sequencing chromatograms showing *sop10* mutations in LKZ1, ZJZ17, and ZZ35.

**Supplementary Table 1**. Primers used in this study

| **Intron retention test** | | | | | | | | | | |
| --- | --- | --- | --- | --- | --- | --- | --- | --- | --- | --- |
| **Primers** | | **Sequences 5′ to 3′** | | | | | | | **Target gene** | |
| CF8597 | | TTCTAATGATCGCCGTGTCC | | | | | \| *nad4* \| \| --- \| \| | | | exon 1 |
| CF8598 | | CGAACCCCATACCCCTATAATG | | | | |  |  |  | exon 2 |
| XP42 | | GTTACCCGAAGCCCATGTAGAGGCACCTAC | | | | | *nad4* | | | exon 2 |
| XP43 | | CCCCAATAAAGCTGCTAGTGCCGGGTAAAC | | | | |  |  |  | exon 3 |
| XP44 | | CTATGTGTTGGTGTTCTATATGACCGACAT | | | | | *nad4* | | | exon 3 |
| CP179 | | TCAATGAAATTTGCCATGTTGCACTAAGTTA | | | | |  |  |  | exon 4 |
| CP680 | | GAACCGCTATAATGACCACC | | | | | *nad5* | | | exon 1 |
| CP681 | | ATGAAGCAAGACCTACTCCC | | | | |  |  |  | exon 2 |
| XP45 | | GAATACTCACCTACGGCTTTGA | | | | | *nad5* | | | exon 2 |
| XP46 | | TACCTAAACCAATCATCATAT | | | | |  |  |  | exon 3 |
| XP47 | | ATATGATGATTGGTTTAGGTA | | | | | *nad5* | | | exon 3 |
| XP48 | | ATCCACTTTGAAGTTGACTTATTC | | | | |  |  |  | exon 4 |
| XP49 | | TTACAAAACTAATACCTATTCTGTTTAG | | | | | *nad5* | | | exon 4 |
| CP181 | | TTATTCTTGACTTGACTTATTAATAAGAAAAC | | | | |  |  |  | exon 5 |
| **Cloning Primers** | | | | | | | | | | |
| **Primers** | | **Sequences 5′ to 3′** | | | | | | **Target gene** | | |
| CF2701 | CACCACCACCACGTGTGAATTGGTGACCTAATGTGGTCAGGGGGGCGTCTGA | | | OsSOP10 UTR3 | | | | | | |
| CF2702 | GTAAAACGACGGCCAGTGCCAAGCTTGGTGTTAGATGGAGGTTTACCTCA | | |  |  |  |  |  |  |  |
| CF2703 | ACAGCTATGACCATGATTACGAATTCGATTCCGGCGAGGTTTAGGGGAGGGA | | | *gOsSOP10* including promoter | | | | | | |
| CF2704 | ACAGATCTCCATGGTCTAGAGGATCCCAAGAATGAATCCTCGAAGGCTCTC | | |  |  |  |  |  |  |  |
| CF7481 | AAGCTTCTGCAGGGGCCCGGGGTCGACACAGGGAAGATGGCGAGCGC | | | *OsSOP10* CDS | | | | | | |
| CF7482 | CTCCTCGCCCTTGCTCACCATGGTACCCAAGAATGAATCCTCGAAGGCTCTCAAGCA | | |  |  |  |  |  |  |  |
| CP796 | ATGACGACGATAAGACTAGTCCCGGGATGGCGCTCCGCACGCTGGCCT | | | Mn-SOD gene (LOC_Os05g25850) | | | | | | |
| CP797 | TGCCTGCAGGTCGACTCTAGAGGATCCTCAAGCAGTCGCATTTTCGTACACCT | | |  |  |  |  |  |  |  |
| CP798 | ATGACGACGATAAGACTAGTCCCGGGATGGCGTTCGCCACACTGGTGGGAGT | | | Fe-SOD gene (LOC_Os06g05110) | | | | | | |
| CP799 | TGCCTGCAGGTCGACTCTAGAGGATCCTCACACCCCTAGGGACTTCTCTTGACCGCT | | |  |  |  |  |  |  |  |
| CP802 | ATGACGACGATAAGACTAGTCCCGGGATGGTGAAGGCTGTTGCTGTGCTTGCTA | | | Cu/Zn-SOD gene (LOC_Os07g46990) | | | | | | |
| CP803 | TGCCTGCAGGTCGACTCTAGAGGATCCCTAACCCTGGAGTCCGATGATTCCGCAA | | |  |  |  |  |  |  |  |
|  |  | | |  | | | | | | |
| **CRISPR/Cas9 sgRNA clone primers** | | | | | | | | | | |
| **Primers** | | **Sequences 5′ to 3′** | | | | | | **Target gene** | | |
| CF2859 | | GGCAGGCGTGGGCGCGGCTGGGCT | sgRNA#1 for *OsSOP10* | | | | | | | |
| CF2560 | | AAACAGCCCAGCCGCGCCCACGCC |  |  |  |  |  |  |  |  |
| CF2861 | | GCCGTAGGAGTAGTCGTTGGGGC | sgRNA#2 for *OsSOP10* | | | | | | | |
| CF2862 | | AAACGCCCCAACGACTACTCCTA |  |  |  |  |  |  |  |  |
| CF2863 | | GTTGCCAGCGTGCTCAGCGCGTG | sgRNA#3 for *OsSOP10* | | | | | | | |
| CF2864 | | AAACCACGCGCTGAGCACGCTGG |  |  |  |  |  |  |  |  |
| CF2859 | | GGCAGGCGTGGGCGCGGCTGGGCT |  | | | | | | | |
| **Genotype analysis** | | | | | | | | | | |
| **Primers** | | **Sequences 5′ to 3′** | **Target gene** | | | | | | | |
| CF4461 | | GTGAGCCCAAATCAACCTTAG | *OsSOP10* CRISPR genotyping | | | | | | | |
| CF4462 | | GCGAGCCATTCCGTAGAACA |  |  |  |  |  |  |  |  |
| **Gene expression analysis** | | | | | | | | | | |
| **Primers** | | **Sequences 5′ to 3′** | | | **Target gene** | | | | | |
| HX6686 | | GCGTGATCTCACTGATTACCTC | | *ACTIN* | | | | | | |
| HX6687 | | ATCAGGAAGCTCGTAGCTCTT | |  |  |  |  |  |  |  |
| CP0983 | | TTCATAGTTGCATTACTTATAGCTTCCT | | Pre-16S rRNA probe | | | | | | |
| CP0985 | | CCTCGCCATTAACGTTAAGGCTATGCCA | | Pre-23S rRNA probe | | | | | | |
| CP2246 | | AGAGAAACGCACAGACCAATCAATCGTATC | | | | *nad4* probe | | | | |
| CP2862 | | ACCTGAAGTACTAAACAGAATAGGTATTAG | | | | *nad5* probe | | | | |
| XP23 | | GAAGTGTCTCTGGGCTCAAG | | | | Cu/Zn-SOD gene (LOC_Os07g46990) | | | | |
| XP24 | | TGGCGGTTCTCATCTTGTG | | | |  |  |  |  |  |
| XP29 | | ATAACCTCAAGCCTATCAGCG | | | | Mn-SOD gene (LOC_Os05g25850) | | | | |
| XP30 | | ACCCATCCAGATCCTTGTAAAG | | | |  |  |  |  |  |
| XP31 | | CGCTCAGCATTATCACTTTTGG | | | | Fe-SOD gene (LOC_Os06g05110) | | | | |
| XP32 | | GTGGACTGATGGCATTTTGTG | | | |  |  |  |  |  |
| **Mitochondrial gene expression analysis** | | | | | | | | | | |
| **Primers** | | **Sequences 5′ to 3′** | | | | **Target gene** | | | | |
| CF8627 | | TTGCCATATCTTCGCTAGGTG | | | | *nad1* | | | | |
| CF8628 | | CAAGGGAATACCGGACCATATC | | | |  |  |  |  |  |
| CF8593 | | TTTTAGCGGTTTCCCCAGAG | | | | *nad2* | | | | |
| CF8594 | | GGGCAATAGTTAGGAGAGGTG | | | |  |  |  |  |  |
| CF8595 | | AATGTGGTTTCGATCCCTCC | | | | *nad3* | | | | |
| CF8596 | | GAGGTACTGCCCAAGGAAAA | | | |  |  |  |  |  |
| CF8597 | | TTCTAATGATCGCCGTGTCC | | | | *nad4* | | | | |
| CF8598 | | CGAACCCCATACCCCTATAATG | | | |  |  |  |  |  |
| CF8599 | | TGGTATTTTCCGTTTCTTTGGATG | | | | *nad4L* | | | | |
| CF8600 | | AATTCGACAGCAATAGTCCCTC | | | |  |  |  |  |  |
| CF8629 | | GCGGCATCTCTAACTATTCGG | | | | *nad5* | | | | |
| CF8630 | | AGACACTTCCCAACCAGAAAG | | | |  |  |  |  |  |
| CF8601 | | TCAGTTTTGTCTAGCCCTGC | | | | *nad6* | | | | |
| CF8602 | | GCTGTGTCGCAAAAGATTAGG | | | |  |  |  |  |  |
| CF8603 | | TGCCAGTTTCATACGACCTG | | | | *nad7* | | | | |
| CF8604 | | GAATCCCCAATCCTTTGCTTG | | | |  |  |  |  |  |
| CF8605 | | TCAGTCCATTTCCATCAGCC | | | | *nad9* | | | | |
| CF8606 | | CGTACTTCCACATATCCACTCAG | | | |  |  |  |  |  |
| CP1946 | | ACCCCTATTCTCCCTTCATCC | | | | LOC_Osm1g00110 | | | | |
| CP1947 | | AAATAGTGGAGGGTGCTTGG | | | |  |  |  |  |  |
| CP1948 | | TTCAAAGAAACTCTCGACGGG | | | | LOC_Osm1g00120 | | | | |
| CP1949 | | GCACTGTCTTTTCGCACTTAG | | | |  |  |  |  |  |
| CP1950 | | TCCAACCAGAATCCGAAACC | | | | LOC_Osm1g00130 | | | | |
| CP1951 | | CACTGTTGACGAATAGAGGACG | | | |  |  |  |  |  |
| CP1952 | | GAAGTGGTCAAAGTAGGAGTCG | | | | LOC_Osm1g00140 | | | | |
| CP1953 | | CGGTAAGCATCCAGTATCTCAG | | | |  |  |  |  |  |
| CP1954 | | TCAAGATCAAGTACCGAAGCAG | | | | LOC_Osm1g00150 | | | | |
| CP1955 | | CAAACCGAATATCCAGCAACC | | | |  |  |  |  |  |
| CP1956 | | CCGTTAGGGAGAATGACTGATG | | | | LOC_Osm1g00160 | | | | |
| CP1957 | | ATCTCACCGTAAATATGCCCG | | | |  |  |  |  |  |
| CP1958 | | TCTCAAACTACGGAACCAACTG | | | | LOC_Osm1g00170 | | | | |
| CP1959 | | GGGATACTTCGGATAAACTGGAG | | | |  |  |  |  |  |
| CP1960 | | TGGGCTGTACAAAGAACTGAC | | | | LOC_Osm1g00180 | | | | |
| CP1961 | | CTGTCAGCGAGAAAATGTTGG | | | |  |  |  |  |  |
| CP1962 | | TCCTATTTCAATCCGTGCTGG | | | | LOC_Osm1g00200 | | | | |
| CP1963 | | CGAACAAGATACGGGTAGAGAAG | | | |  |  |  |  |  |
| CP1964 | | CTGATGCTGTGACCTATGAGAG | | | | LOC_Osm1g00210 | | | | |
| CP1965 | | TGGGTGAATGGAGAATGGATC | | | |  |  |  |  |  |
| CP1966 | | TTTCGATCCCCAGTTGCTATG | | | | LOC_Osm1g00220 | | | | |
| CP1967 | | GTACCTGAGAGCATACCGATG | | | |  |  |  |  |  |
| CP1968 | | GATGTTGGTTCGCGCTTTATG | | | | LOC_Osm1g00240 | | | | |
| CP1969 | | AAGGCGGAATTTGAGGGTC | | | |  |  |  |  |  |
| CP1970 | | TGAGACTCTTGCTTCCCTTTG | | | | LOC_Osm1g00250 | | | | |
| CP1971 | | ACTCACCTTCATCCACCTTTC | | | |  |  |  |  |  |
| CP1972 | | AGATCCAAGTCGGTTCAATG | | | | LOC_Osm1g00260 | | | | |
| CP1973 | | CTTTCCCAAATGCCCACCA | | | |  |  |  |  |  |
| CP1974 | | GCACCAAAAGTTGTAGAGCTG | | | | LOC_Osm1g00270 | | | | |
| CP1975 | | TCCTTTTCCTCTTCCCATTCG | | | |  |  |  |  |  |
| CP1976 | | CAATGTCCCCAAAAGCAAGG | | | | LOC_Osm1g00290 | | | | |
| CP1977 | | GCGAATCTTTCACTCTACCTCC | | | |  |  |  |  |  |
| CP1978 | | CCCCGTTTTGACCCTTATTC | | | | LOC_Osm1g00300 | | | | |
| CP1979 | | AAGTTGAGGTTCAGGGTTCG | | | |  |  |  |  |  |
| CP1980 | | TTTCTACAATCCCGGAACAGG | | | | LOC_Osm1g00310 | | | | |
| CP1981 | | GATTTGTTCCCCTACTCTCCG | | | |  |  |  |  |  |
| CP1982 | | TCCAGCCATTACTATCAAA | | | | LOC_Osm1g00330 | | | | |
| CP1983 | | GGAAGGTACAGCCCAACTA | | | |  |  |  |  |  |
| CP1984 | | AGTTATAGATGGTTGCCGTTCG | | | | LOC_Osm1g00340 | | | | |
| CP1985 | | GTTTCAATTTGGGCAAGAGGG | | | |  |  |  |  |  |
| CP1986 | | GCACTCTTTTCCACCCTATCC | | | | LOC_Osm1g00350 | | | | |
| CP1987 | | TGCCTTCTCTTTCTGTCTTCC | | | |  |  |  |  |  |
| CP1988 | | GTACCAAATGCCGAATTAGCG | | | | LOC_Osm1g00360 | | | | |
| CP1989 | | ATCTTCGTCCTACCAAAACCG | | | |  |  |  |  |  |
| CP1990 | | ATGAATCCTTGTCTATGGCGG | | | | LOC_Osm1g00370 | | | | |
| CP1991 | | AGTAAAAGTGACCGAGATGCG | | | |  |  |  |  |  |
| CP1992 | | GTTTTGTCAACTCGCCTGAAG | | | | LOC_Osm1g00380 | | | | |
| CP1993 | | GACCTTGTATCATTGGCTTGC | | | |  |  |  |  |  |
| CP1994 | | AGCCATTCAGCTTCGTTATCG | | | | LOC_Osm1g00390 | | | | |
| CP1995 | | AAGGGCGATCCATCTTGATG | | | |  |  |  |  |  |
| CP1996 | | TTCCGCTTACTCACAACTCAG | | | | LOC_Osm1g00400 | | | | |
| CP1997 | | CCGCTTCCCCTATTGTTCTTATC | | | |  |  |  |  |  |
| CP1998 | | AATAAAAGGGCGGTCTCGTC | | | | LOC_Osm1g00410 | | | | |
| CP1999 | | GATCTCTTACTCTTTCACCCCG | | | |  |  |  |  |  |
| CP2000 | | AAAGTGCCCTATGTCTCGC | | | | LOC_Osm1g00420 | | | | |
| CP2001 | | CCGTGGAGAAAGACCGTATTC | | | |  |  |  |  |  |
| CP2002 | | TGCTGTCGGTATTGGAAACG | | | | LOC_Osm1g00430 | | | | |
| CP2003 | | GTGAGAGCAAAGCCCAAAATG | | | |  |  |  |  |  |
| CP2004 | | AGGATGGAGCGTACAAAGTCG | | | | LOC_Osm1g00440 | | | | |
| CP2005 | | TGGGCAGGTCGCAAGAAGC | | | |  |  |  |  |  |
| CP2006 | | GCAGGAAAATTTGGTCACGTAG | | | | LOC_Osm1g00450 | | | | |
| CP2007 | | GTTTGATAGGGTCTTCGTCTCC | | | |  |  |  |  |  |
| CP2008 | | CTCAGTCTATTGTGCAGCTAGG | | | | LOC_Osm1g00470 | | | | |
| CP2009 | | GGTCCCAAGGTAGAAAGTGAAG | | | |  |  |  |  |  |
| CP2010 | | TGTTTGGTGTCTCGGAGTTG | | | | LOC_Osm1g00480 | | | | |
| CP2011 | | CGATTTAAGGTGGCATTGTCC | | | |  |  |  |  |  |
| CP2012 | | CTCCGTCCCAAACAGTGTAC | | | | LOC_Osm1g00490 | | | | |
| CP2013 | | CTCTTTACCCTCCCCACTATG | | | |  |  |  |  |  |
| CP2014 | | AGAATGTAGTGGCTGGTGAATC | | | | LOC_Osm1g00500 | | | | |
| CP2015 | | CGGTTTTGTCTGAATCTTTGTGG | | | |  |  |  |  |  |
| CP2016 | | CTTTCCTATCAGCGAGTTCCG | | | | LOC_Osm1g00510 | | | | |
| CP2017 | | GCAGCGTTCCATTCTTTTCG | | | |  |  |  |  |  |
| CP2018 | | GTGTGCATCTTTATGTTGGGTG | | | | LOC_Osm1g00520 | | | | |
| CP2019 | | AATGGAATGAGAAGTGGGTCC | | | |  |  |  |  |  |
| CP2020 | | TCTTCCGTGATGTGGTTTCC | | | | LOC_Osm1g00530 | | | | |
| CP2021 | | TTGCTTGGGCTCGTTCTATC | | | |  |  |  |  |  |
| CP2022 | | AGGGAGTTCACTGGTCAAAC | | | | LOC_Osm1g00540 | | | | |
| CP2023 | | AGAGATGGATAGAGGCGAGAG | | | |  |  |  |  |  |
| CP2024 | | CGTGGACCTGGAATGACTATG | | | | LOC_Osm1g00550 | | | | |
| CP2025 | | GAATATACACCTCTGGATGACCG | | | |  |  |  |  |  |
| CP2026 | | AGAGTTCAATGGCACGAGTC | | | | LOC_Osm1g00560 | | | | |
| CP2027 | | TGCCCATCAAAGAACCTCG | | | |  |  |  |  |  |
| CP2028 | | GCGGTCAGAGATTCATACAGAC | | | | LOC_Osm1g00570 | | | | |
| CP2029 | | TTTCCCATATTTCGACCGGAG | | | |  |  |  |  |  |
| CP2030 | | GCGGAACTCACGACTCTATTAG | | | | LOC_Osm1g00580 | | | | |
| CP2031 | | TGCAATCCCATCTCCAACTG | | | |  |  |  |  |  |
| CP2032 | | TTATTTCTCCGTGCCCCTTC | | | | LOC_Osm1g00600 | | | | |
| CP2033 | | CAGCCCTTTCCATTTTCGTTG | | | |  |  |  |  |  |
| CP2034 | | CAAGTCCCTGCCATTTTGAAC | | | | LOC_Osm1g00610 | | | | |
| CP2035 | | AGAGACACTCGACAAAAGACG | | | |  |  |  |  |  |
| CP2036 | | CGTGTTTTCTGTGCTTTTCCC | | | | LOC_Osm1g00620 | | | | |
| CP2037 | | GAAGTGGTTGGATTTTGCGAG | | | |  |  |  |  |  |
| CP2038 | | TCCGTGAATGTGAGGACTTTG | | | | LOC_Osm1g00630 | | | | |
| CP2039 | | TGGAACGTGATGGGATTGTC | | | |  |  |  |  |  |
| HX6686 | | GCGTGATCTCACTGATTACCTC | | | | qRT-PCR for rice *ACTIN* | | | | |
| HX6687 | | ATCAGGAAGCTCGTAGCTCTT | | | |  |  |  |  |  |
| **RIP RT-PCR** | | | | | | | | | | |
| CP4627 | | CGTGCTAGGAAGCATTA | | | | *nad4* | | | | |
| CP4628 | | AGACCAACCCACTGAAA | | | |  |  |  |  |  |
| CP4633 | | GCCGTCAAAGTGCCTAT | | | | *nad4* | | | | |
| CP4634 | | TTCGGACTAAACATACCAAT | | | |  |  |  |  |  |
| CP4641 | | TGGGATGGGAGGGAGTAG | | | | *nad5* | | | | |
| CP4642 | | ATCGGGTAACCAAGTATGCA | | | |  |  |  |  |  |
| CP4643 | | AGGGTCCCACTCCAGTAT | | | | *nad5* | | | | |
| CP4644 | | GAAAGACGCTAACCGAAT | | | |  |  |  |  |  |
| CF2274 | | CGTCTTGCCAGGTTATCATCTA | | | | *ACTIN* | | | | |
| CF2275 | | ATTTTGACTAAGTTCAGGCTTT | | | |  |  |  |  |  |

**Supplementary Table 2.** Primer sets for RT-PCR for amplifying mitochondria-encoded protein coding transcripts.

| Gene | Primer set (sequence direction is 5’ to 3’) |
| --- | --- |
| *nad1* | nad1-EDF (ATGATTGGGTCAACCAGGCC)  nad1-R1 (GAAACTTCCTGGCACATACA) |
| *nad2* | nad2-newF (CCTGCTGCTCATGGTGTTTC)  nad2-newR1 (CCACTAGATTGAGCACCAGT) |
|  | nad2-F1je (GTCTGAATTTTCCACGGAAG)  nad2-R1 (CACGGGTTTGCCGTAATGCT) |
|  | nad2-F2 (ACGATAGATGCATTCGCCAT)  nad2-newR2 (TAGTGCTTACCTGCAGTCCA) |
| *nad3* | nad3-EDF (AAGGACAAGATTGAGTCGCC)  nad3-EDR (GGCATCCCTCTTTCCTATGT) |
| *nad4* | nad4-F1 (GATTGACTGTTGTCAACTAATC)  nad4-R1 (CTGATATGCTGCCTTGATCT) |
|  | nad4-F2 (GTATGGGGTTCGAGACAAAG)  nad4-R2 (CAAGTCGAGTCTTATGTCGG) |
|  | nad4-F3 (GTTAAGTCATGGACTGGTTTC)  nad4-R3 (GCCATGTTGCACTAAGTTAC) |
| *nad4L* | nad4L-EDF (GACTCTACGCAGGACTTCTT )  nad4L-EDR (TAACTCCCCTGTAGTTATGT) |
| *nad5* | nad5-F (GTAGGATACTTGGCCAAAGA)  nad5-newR1 (GGCAAGCTCCTACAGTTCTC) |
| *nad6* | nad6-F (CTCGAACCACAATTCTCCTT)  nad6-R (CCATTTCAAGGGAGGACGAC) |
|  | nad6-Fnew (GGTCTGTCGTCCTCCTCATT)  nad6-Rnew (GATACTTTCAGTTTTGTCTAGC) |
| *nad7* | nad7-F1 (ATGACGACTAGGAACGGGCA)  nad7-R1 (CTGTGCAGTGACAGTACCAA) |
|  | nad7-F2 (CTGGAAACAACGATTAGTGG)  nad7-R2 (GATCGAGCAAGACTACGTC) |
| *nad9* | nad9-EDF (CTGCTTAGAGCAAGAAGCG)  nad9-EDR (CTAGTTATGTAGATTCGGAA) |
| *cox1* | cox1-F (GACGTTGATACGCGTGCCT)  cox1-R (GATAGCTGGAAGTTCTCCAA) |
| *cox2* | cox2-F (CAGTCTCCTTTCTAGGAGCA)  cox2-R (TGCATTTCCGCTTCAGCTTC) |
| *cox3* | cox3-F0 (GAAGAGCCTCCTTCTTTACC)  cox3-R0 (CCACTTATTCGTTCCCTTCT) |
|  | cox3-F (CATAGTCTCGGAGGTTATGT)  cox3-newR (CCACTTATTCGTTCCCTTCT) |
| *atp1* | atp1-F1 (GGAATTCTCACCCAGAGCT)  atp1-R1 (CCAGTTTGTCTGTCCCCGA) |
|  | atp1-F2 (AGAGCCGCTAAACGATCGGA)  atp1-R2 (CTAATTAATCTCCTTCGCAGTT) |
| *atp4* (*orf25*) | atp4-F (GGGATTGAGTTCAACGGATA)  atp4-R (CAAGCCTTCCCGAATTAGGT) |
| *atp6* | atp6-F2 (GGGAAATTCCAATGAATTTCG)  atp6-R2 (GTTTACCAGGTTCAGCACGA) |
|  | atp6-F3 (TCGTGCTGAACCTGGTAAAC)  atp6-R3 (CGGCTCCTCGTTTTTATTCA) |
| *atp8* (*orfB*) | atp8-F1 (CACAATTCTTCTGGTTATGCC)  atp8-R1 (TATGCTTCCTTGCCCGTGT) |
| *atp9* | atp9-F (AGCGTGACGAGCAAAGTCAA)  atp9-R (CCTCGGTATCTCTATTTGCA) |
| *ccb2* (*ccmB*) | ccb2-F (CCAGCCGTCGAAGTGAATGA)  ccb2-R (TCCATGACTTGGCCATTCAA) |
| *ccb3* (*ccmC*) | ccb3-F (GCTACGCGCAAATTCTCATTG)  ccb3-R (CGAGCTTCTATTTCTTCCGT) |
| *ccb6c* (*ccmF_C_*) | ccb6c-F1 (GGTCCAACTACAGAACTTCT)  ccb6c-R1 (CTTCAAGCCCGATTTCAGGT) |
|  | ccb6c-F2 (GCTCTCGCCTTACCAACGA)  ccb6c-R2new (GCAATTATGAACGAAACTTTCTC) |
| *ccb6n* (*ccmF_N_*) | ccb6n-F (GGCTTTGGGTTATGTAGATC)  ccb6n-R (GCCTCCTGCTTCATCTGGTA) |
|  | ccb6n-F2 (GGAGATTGGGATCATCCTGT)  ccb6n-R2 (GTGGAGTGCCACAATCCCAT) |
| *cytb* (*cob*) | cytb-F (CCAACTCCGAGCAATCTTAG)  cytb-R (GATCACTGATCAGGTGTGAT) |
| *orfX* | orfx-F (CTCATTGAATGGAATTTCGCA)  orfx-R (GTCCAGCCCTCTTCACGAA) |
| *pseudo-rpl16* (*rpl16*) | rpl16-F (GCGGAAATAGCTAGAACTGA)  rpl16-R (CCACTAACCAATTACGTTACG) |
| *pseudo-rps11* | rps11-F (CGTCAACACTATTCACATAGG)  rps11-R (GGTCACATCGTGGATGTACA) |
| *rpl2* | rpl2-F (CCAAGAGCTTGGACGCACA)  rpl2-R (GAAGGTCTACCTCCTTTCGT) |
| *rpl5* | rpl5-F (CGTCAGGATCTGTTGCTCAA)  rpl5-R (CCGAAGTGACAATAGTCACA) |
| *rps1* | rps1-F (GTGGATGCAGGCCTTGGTA)  rps1-R (GATTAAGGTGGGCTTCGGA) |
| *rps2* | rps2-F (CGAAATAGCTCAGTTCGAGA)  rps2-R (GTAGCGCTACAGATTGAAGT) |
| *rps3* | rps3-F1 (GGTAAGACTTGATCTGAATCG)  rps3-R1 (CGACGGGGTCGAAATGCAT ) |
|  | rps3-F2 (CCAAGCGAATGATCTTCGCT)  rps3-R2 (CGGATATAGCACGTCCCTT ) |
| *rps4* | rps4-F (GGAAGAGTTGGGTTCGATTC  rps4-R2 (GTAAGCGGAACCATTCTGTT) |
|  | rps4-F2 (GGCAAATTACTACCGGTCA)  rps4-R3 (GTTTTGGCCACGTCCGTTTC) |
| *rps7* | rps7-F (GGGACTTTGATGGTGAGC)  rps7-R (CTTTCGGTAAGCATCCAGTA) |
| *rps13* | rps13-F (GTAGATTCCAGCCGAGAAGA)  rps13-R (TCCGAATTAGCTTGCGAGCA) |
| *rps19* | rps19-F (GCCACGACGATCTATATGGA)  rps19-R(CCGAATATGTCGCTTAGACT) |

**Uncropped scans of Northern blots for Supplementary Fig. 2**

MB staining and Pre-16S rRNA Northern Blot (source data for **Supplementary Figure 2b**)


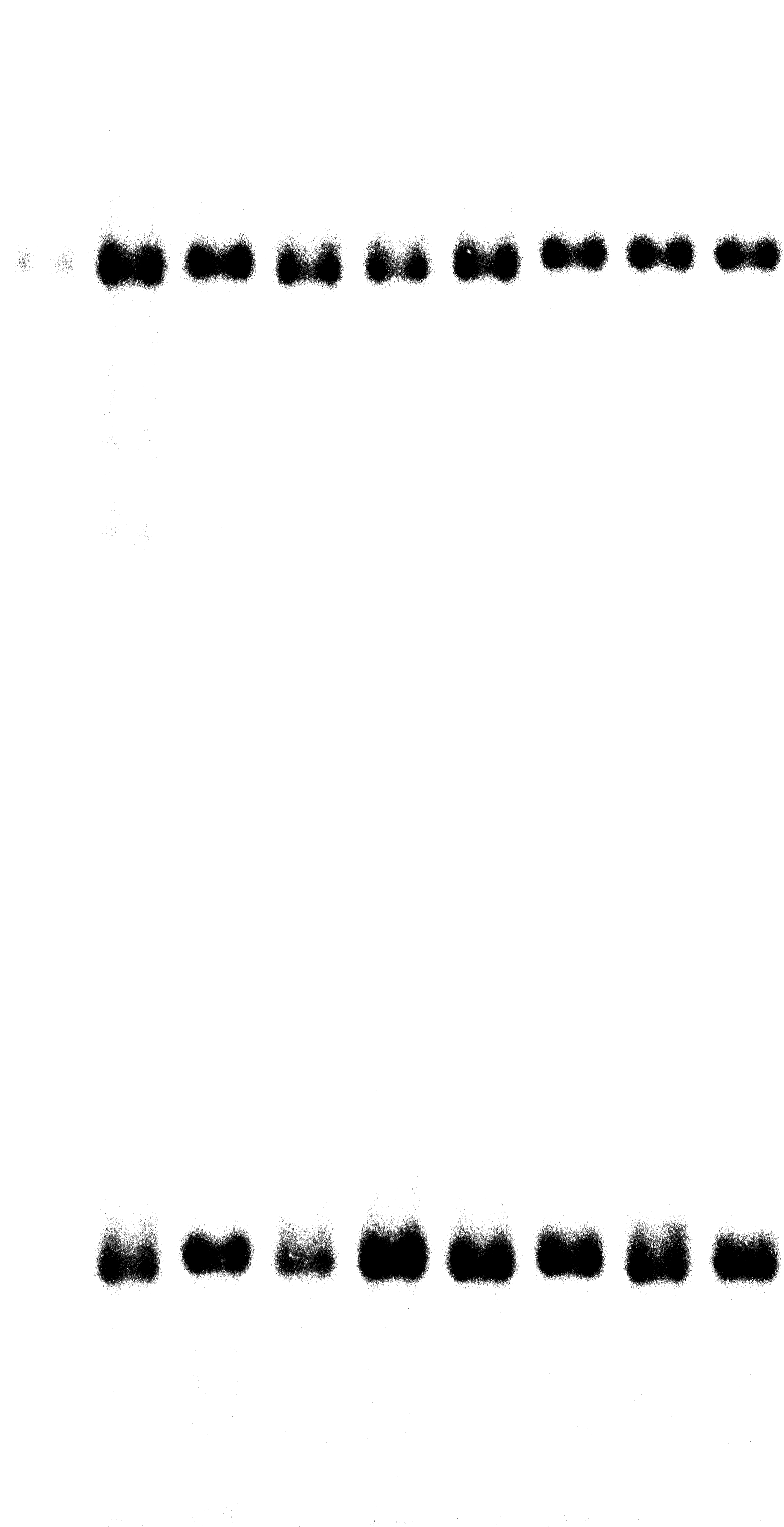

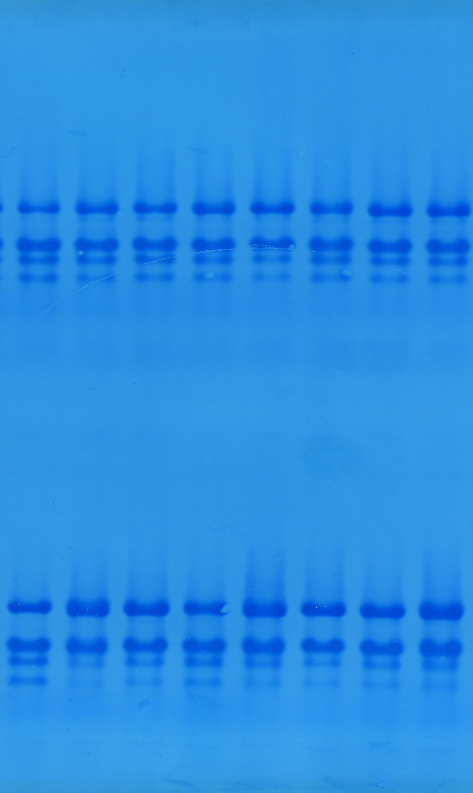


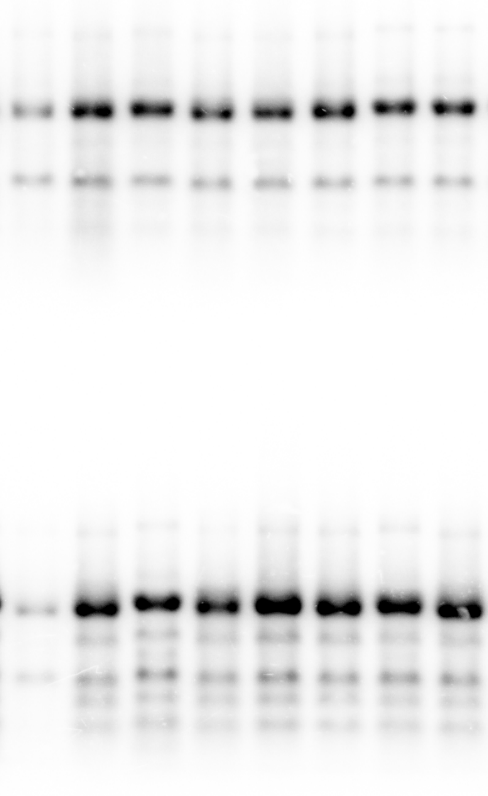

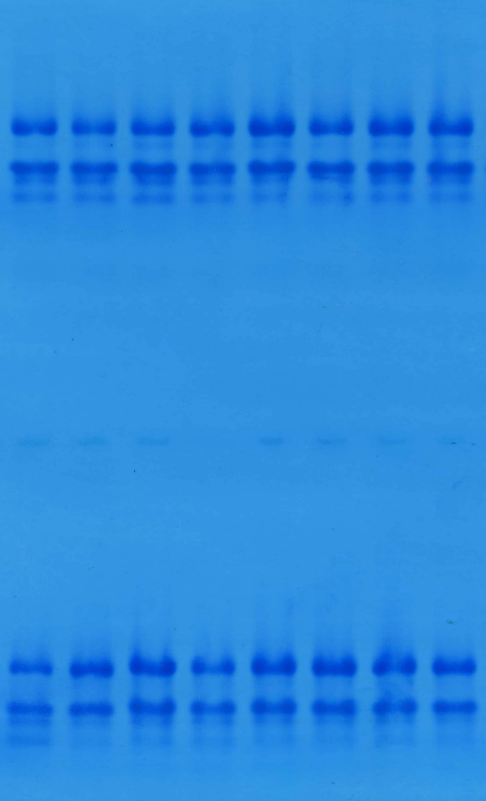
MB staining and Pre-23S rRNA Northern Blot (source data for **Supplementary Figure 2c**)

**Uncropped scans of Blue Native PAFE gel for Supplementary Fig. 7b**


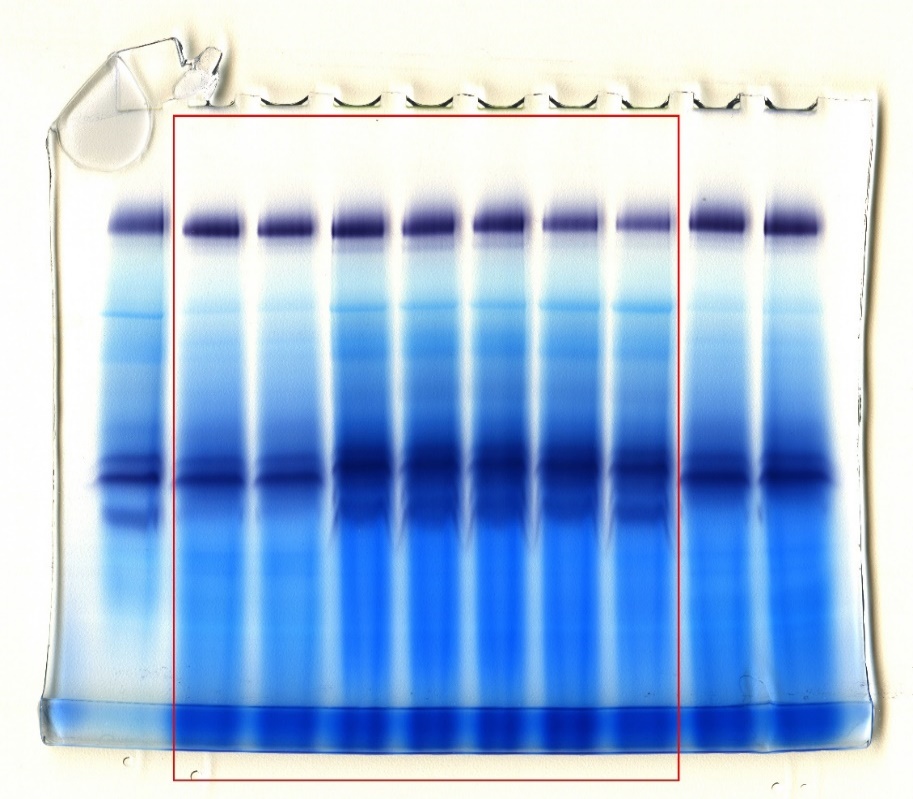

Supplement: Supplementary file 1 — Supplementary Information [file 41467_2023_42269_MOESM1_ESM.docx]
