# Supplementary material for: A mitochondrial pentatricopeptide repeat protein enhances cold tolerance by modulating mitochondrial superoxide in rice
Source: Nat Commun. 2023 Oct 25;14:6789. doi: 10.1038/s41467-023-42269-4 (PMC10600133; doi:10.1038/s41467-023-42269-4)
Supplement: Supplementary file 3 — Description of Additional Supplementary Files [file 41467_2023_42269_MOESM3_ESM.docx]

**Legends for Supplementary Data**

**Supplementary Data 1 (supports Figure 2b)**. Relative expression levels of 56 mitochondrial genome encoded genes in 9311, *ospus1-1* and *ospus1-1 sop10-1* plants grown at 28°C or 22°C. The total RNA was extracted from above-ground parts from 9311, *ospus1-1* and *ospus1-1* *sop10-1* rice seedlings at 12 DAP. Each RNA sample was extracted from six independent seedlings (*n* = 6), three biological replicates were used for analyzing the expression level of all the 56 mitochondrial genes by RT-qPCR. The quantification data of relative expression level for each gene were exported from the CFX Manager software.

**Supplementary Data 2 (supports Supplementary Figure 6)**. Number of reads at each editing site for each library. The 547 C-to-U editing sites in 34 protein-coding mitochondrial transcripts were analyzed in 9311 and *sop10-2* with two independent biological replicates. POS: the CDS position of the C-to-U editing sites in each transcript. C: number of unedited reads, T: number of edited reads, editing %: T/(T+C)%. The credible reads when QUAL ≥300. The average editing efficiency is calculated from two independent biological replicates.
